# Genome Sequence of the Melanin-Producing Extremophile *Aeromonas salmonicida* subsp. *pectinolytica* Strain 34mel^T^

**DOI:** 10.1128/genomeA.00675-13

**Published:** 2013-09-12

**Authors:** M. Elisa Pavan, Esteban E. Pavan, Nancy I. López, Laura Levin, M. Julia Pettinari

**Affiliations:** Departamento de Química Biológica, Facultad de Ciencias Exactas y Naturales, Universidad de Buenos Aires, Buenos Aires, Argentinaa; Biomedical Technologies Laboratory, Department of Electronics, Information and Bioengineering, Politecnico di Milano, Milan, Italyb; IQUIBICEN-CONICET, Facultad de Ciencias Exactas y Naturales, Universidad de Buenos Aires, Buenos Aires, Argentinac; Departamento de Biodiversidad y Biología Experimental, Facultad de Ciencias Exactas y Naturales, Universidad de Buenos Aires, Buenos Aires, Argentinad

## Abstract

The genome of *Aeromonas salmonicida* subsp. *pectinolytica* strain 34mel^T^, isolated from a heavily polluted river, contains several genomic islands and putative virulence genes. The identification of genes involved in resistance to different kinds of stress sheds light on the mechanisms used by this strain to thrive in an extreme environment.

## GENOME ANNOUNCEMENT

*Aeromonas salmonicida* subsp. *pectinolytica* strain 34mel^T^ is an extremophile isolated from the water of a heavily polluted river that receives the effluents of hundreds of tanneries and other industries, as well as urban sewage and fuel hydrocarbons (1). This strain produces abundant melanin and is highly resistant to heavy metals and other pollutants.

Phenotypic characteristics, DNA-DNA hybridization, and 16S rRNA gene sequence analysis determined the classification of 34mel as the type strain of *A. salmonicida* subsp. *pectinolytica*, a new subspecies of *A. salmonicida* (2). Genetic information for this subspecies is scarce and mostly restricted to 16S rRNA genes and a few other sequences. Multilocus sequence typing showed that this subspecies is the most phylogenetically distant among the five subspecies of *A. salmonicida* (3).

The sequence was obtained using a whole-genome shotgun strategy with a Roche 454 GS FLX Titanium pyrosequencer at INDEAR, Argentina, achieving ~22-fold coverage. Assembly was done using Newbler version 2.6 and generated 309 contigs, the largest of which has 327,967 bases. The draft genome is 4,774,629 bases in length, with a mean G+C content of 58.47%.

Annotation was performed using the NCBI Prokaryotic Genomes Automatic Annotation Pipeline (PGAAP) and Rapid Annotations using Subsystems Technology (RAST) (4), revealing 4,245 predicted coding regions and 100 RNA genes, including one 16S rRNA gene. The annotation covered 525 RAST subsystems, including 54% of the coding sequences, and classified 1,085 open reading frames (ORFs) as hypothetical proteins.

Whole-genome analysis using IslandViewer (5) revealed the presence of 12 genomic islands (GI). The largest GI could be related to environmental adaptability, as it contains a mercury resistance operon. This operon has previously been found in plasmids and in GIs in both environmental and pathogenic bacteria (6, 7).

Apart from the mercury resistance genes, many others that could allow strain 34mel^T^ to cope with toxic compounds were found, such as those involved in resistance to chromium, molybdenum, copper, cobalt, arsenic, zinc, and cadmium, including genes coding for several efflux pumps. Many genes involved in responses to other kinds of environmental stress were also detected, such as those related to oxidative stress: genes coding for catalases, superoxide dismutases, peroxidases, and alkylhydroperoxidases.

A relevant characteristic of *A. salmonicida* subsp. *pectinolytica* is its production of large amounts of melanin. Enzymes involved in the homogentisate pathway were found in the genome of 34mel^T^ along with other gene products that might be related to melanin biosynthesis (8, 9). Pectinases, amylases, proteases, and other enzymes that might be of interest for biotechnological applications were also identified.

Although many *A. salmonicida* strains are well-known fish pathogens, there are no reports describing pathogenicity for *A. salmonicida* subsp. *pectinolytica*. However, a search for virulence-related genes in 34mel^T^ yielded genes coding for hemolysins, toxins, chitinases, a collagenase, and adhesion proteins, among others.

The analysis of the genome of *A. salmonicida* subsp. *pectinolytica* will contribute to understanding the lifestyle of this bacterium that inhabits an extremely polluted environment.

### Nucleotide sequence accession numbers.

This whole-genome shotgun project has been deposited at DDBJ/EMBL/GenBank under the accession no. ARYZ00000000. The version described in this paper is version ARYZ01000000.
